# Opportunistic infections complicating immunotherapy for non‐small cell lung cancer

**DOI:** 10.1111/1759-7714.13422

**Published:** 2020-04-20

**Authors:** Ziwei Liu, Tao Liu, Xiaotong Zhang, Xiaoyan Si, Hanping Wang, Jingjia Zhang, Hui Huang, Xuefeng Sun, Jinglan Wang, Mengzhao Wang, Li Zhang

**Affiliations:** ^1^ Department of Pulmonary and Critical Care Medicine, Peking Union Medical College Hospital Peking Union Medical College & Chinese Academy of Medical Sciences Beijing China; ^2^ Department of Clinical Laboratory, Peking Union Medical College Hospital Peking Union Medical College & Chinese Academy of Medical Sciences Beijing China

**Keywords:** Opportunistic infection, immunotherapy, NSCLC

## Abstract

Immunotherapy has produced durable responses in numerous advanced and metastatic cancers, especially advanced non‐small cell lung carcinoma (NSCLC). However, opportunistic infection has become a major risk for patients who have received immune checkpoint inhibitors (ICIs). Early diagnosis of infection is difficult due to an acute disease course and heterogeneity in clinical manifestation. We retrospectively analyzed four cases with NSCLC who received ICIs and developed opportunistic infections. Two of our cases received antecedent glucocorticoids to treat immune‐related adverse events (irAEs), whereas immunosuppressive agents were not used beforehand in the other cases. We highlight that opportunistic infections complicating immunotherapy can be severe and even fatal. When patients deteriorate after initial remission from irAEs by glucocorticoids, infections should be thoroughly investigated and carefully distinguished from an irAE flare. Bronchoscopy and bronchoalveolar lavage (BAL) are essential. In patients where limited results from traditional microbiological tests have been obtained, next‐generation sequencing (NGS) of BAL fluid is beneficial in guiding a precise antimicrobial treatment. An antipneumocystis prophylaxis may also be considered in selected patients.

## Introduction

Immune checkpoint inhibitors (ICIs) have produced durable responses in numerous advanced and metastatic cancers, especially advanced non‐small cell lung carcinoma (NSCLC).^1^ The mechanism of ICIs is to promote reactivation of antitumor immunity. Importantly, infections have become a major risk for patients who receive ICIs.^2^ In particular, for patients who develop immune‐related adverse events (irAE), concurrent use of systemic glucocorticoids further increase the susceptibility to severe infections leading to fatal outcomes.^3^ In our study, we aim to share our experiences and thoughts from four cases with NSCLC who received ICI treatment and developed opportunistic infection.

## Methods

Patients with NSCLC who developed pneumonitis combining opportunistic infections during immunotherapy were retrospectively recruited from Peking Union Medical College Hospital. Chest computed tomography (CT) scans and bronchoscopy were performed to diagnose pneumonitis. Cytological classification, pathogen culture and next generation screening of bronchoalveolar lavage were performed to confirm infections.

## Results

Four male patients with a mean age of (60 ± 2.9) years were analyzed. Clinical and laboratory data of the patients are presented in Table 1.

**Table 1:** Clinical and laboratory data of patients with opportunistic infections complicating immunotherapy for NSCLC

	Age	Gender	Peak BT, °C	WBC, per mm3†	Lymphocytes, per mm3‡	BALF lymphocytes, %	BALF neutrophils, %	BALF pathogens (traditional laboratory tests)	BALF NGS
Case 1	55	Male	38.5	1620	400	1	0	Negative	*Pneumocystis jirovecii*, *Aspergillus fumigatus*, Cytomegalovirus
Case 2	61	Male	38.0	13 800	1040	1	0	*Corynebacterium striatum*, *Candida albicans*	*Corynebacterium striatum*, *Candida albicans*, *Aspergillus fumigatus*, *Alloprevotella tannerae*, *Rothia mucilaginosa*
Case 3	62	Male	38.8	8800	551	N/A	N/A	*Pneumocystis jirovecii* DNA, Cytomegalovirus DNA	*Pneumocystis jirovecii*, Cytomegalovirus
Case 4	62	Male	38.8	9560	1160	6	60	Negative	*Pseudomonas aeruginosa*, *Pneumocystis jirovecii*, and *Candida albicans*

NSCLC, non‐small cell lung cancer; BT, body temperature; WBC, white blood cell; BALF, bronchoalveolar lavage fluid; NGS, next‐generation sequencing.

† Normal range of WBC: 3500–9500 per mm3.

‡ Normal range of lymphocytes: 800–4000 per mm3.

### Case 1

**Figure 1:**
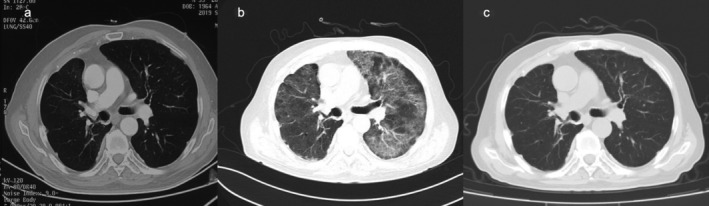
Computed tomography (CT) scan findings in Case 1. **(a**) Stable disease after two cycles of nivolumab and docetaxel. (**b**) Diffuse ground‐glass opacities (GGOs) were visible bilaterally. (**c**) Radiographic lesions resolved markedly after antimicrobial and glucocorticoid treatment.

A 55‐year‐old patient with lung squamous carcinoma and diabetes mellitus complained of fever and dry cough for one week. He had been evaluated as stable disease after three cycles of nivolumab (Opdivo; Bristol‐Myers Squibb; 300 mg) (Fig 1a). On presentation, partial pressure of arterial oxygen (PaO_2_) was 47 mmHg. Serum CMV‐DNA was 500 copies/mL. Serum (1‐3)‐ß‐d‐glucan level was 2238.8 pg/mL, and galactomannan was 0.41 μg/L. Chest CT scans showed diffuse ground‐glass opacities (GGOs) (Fig 1b). Bronchoscopy revealed massive purulent secretion throughout large airways. The percentage of lymphocytes in bronchoalveolar lavage fluid (BALF) was 1%. Next‐generation sequencing (NGS) assay of BALF identified *Pneumocystis jirovecii*, *aspergillus fumigatus*, *and* cytomegalovirus (CMV). Trimethoprim‐sulfamethoxazole (TMP‐SMX) (20 mg/kg/day), caspofungin (50 mg per 12 hours), and (5 mg/kg per 12 hours) were initiated. Intravenous methylprednisolone was given concurrently (40 mg per 12 hours for five days→40 mg/day for three days). The symptoms and radiographic lesions of the patient alleviated markedly (Fig 1c).

### Case 2

**Figure 2:**
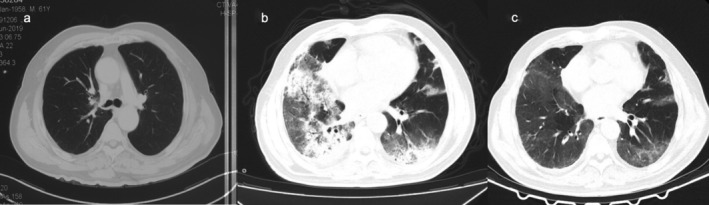
Computed tomography (CT) scan findings in Case 2. (**a**) Stable disease after six cycles of paclitaxel, carboplatin and pembrolizumab. (**b**) Extensive ground‐glass opacities (GGOs) and multifocal consolidations were evident. (**c**) Radiographic lesions resolved markedly after antimicrobial and glucocorticoid treatment.

A 61‐year‐old male with advanced lung adenocarcinoma and diabetes mellitus was admitted to hospital due to acute onset of fever, dyspnea, and productive cough for two days. The patient was treated with paclitaxel, carboplatin and pembrolizumab (Keytruda; Merck Sharp & Dohme; 200 mg) for six cycles (Fig 2a). Three cycles of 200 mg pembrolizumab were then administered, and he received the last dose four days prior to admission. On presentation, chest CT showed extensive GGOs and multifocal consolidations (Fig 2b). Intravenous methylprednisolone was administered (1.0 mg/kg/day) empirically, and his symptoms improved slightly. Bronchoscopy with bronchoalveolar lavage (BAL) was performed. Percentage of lymphocytes in bronchoalveolar lavage fluid (BALF) was 1%. Pathogen culture of BALF identified Corynebacterium striatum and *Candida albicans*. NGS of BALF found Corynebacterium striatum. Vancomycin (1 g per 12 hours) and caspofungin (75 mg once→50 mg per 12 hours) were initiated accordingly. The patient clinically and radiographically improved (Fig 2c).

### Case 3

**Figure 3:**
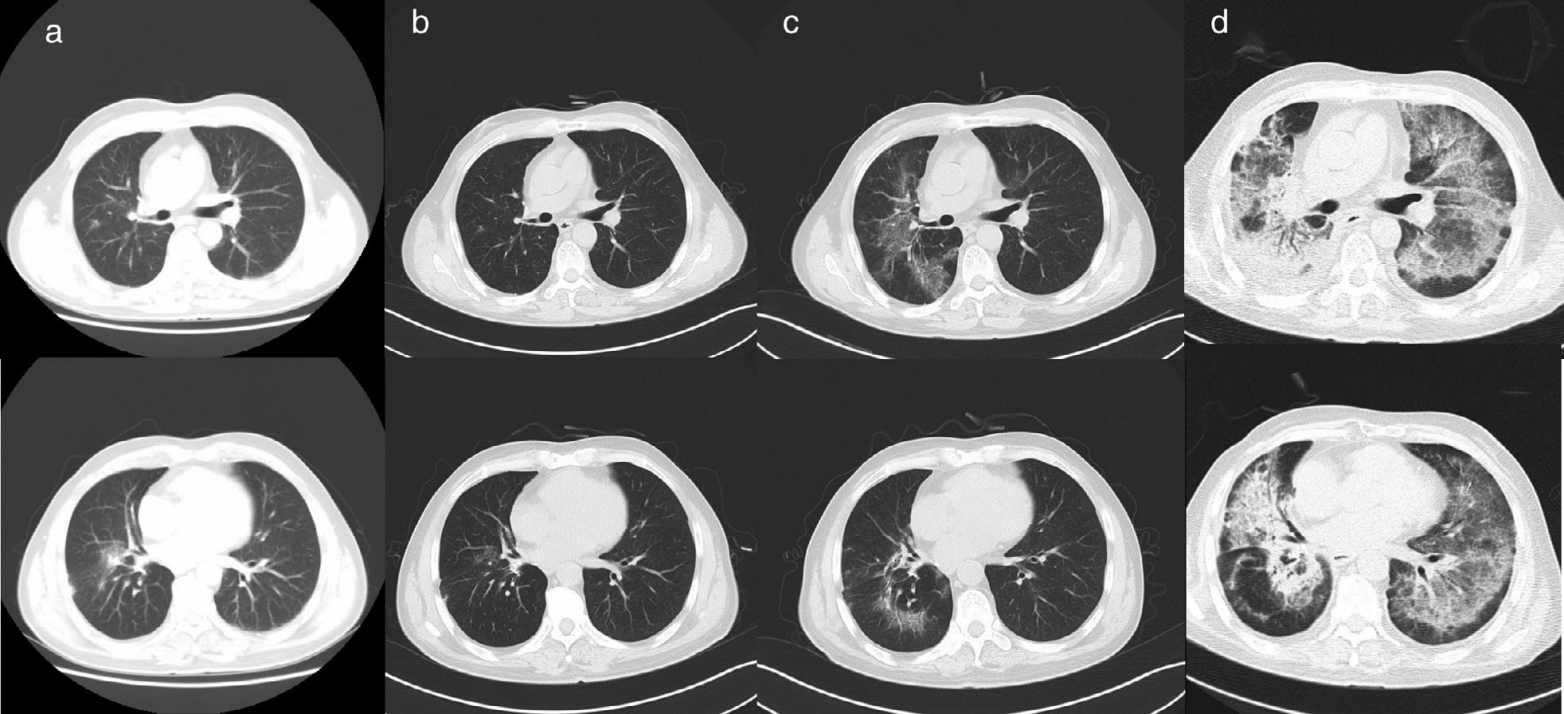
Computed tomography (CT) scan findings in Case 3. (**a**) Multiple irregular opacities on the right lung and radiation pneumonia were suspected. (**b**) There was remission of radiation pneumonia after steroid treatment. (**c**) Ground‐glass opacities (GGOs) were evident when the patient developed fever. (**d**) There were extensive ground‐glass opacities (GGOs) and multifocal consolidations bilaterally.

A 62‐year‐old male with squamous lung carcinoma presented with fever, cough, and hemoptysis for four weeks. The patient had undergone a right lower lobectomy in 2015. A recurrence was found in the residual lung at the surgical site. He completed radiotherapy, then received two cycles of pembrolizumab (Keytruda; Merck Sharp & Dohme; 100 mg). Three weeks after the last dose of pembrolizumab, CT scans indicated multiple irregular opacities on the right lung (Fig 3a). Radiation pneumonia was suspected. The patient received intravenous methylprednisolone 80 mg/day for 10 days, and was clinically and radiographically improved (Fig 3b). His steroid dose was tapered gradually. Unfortunately, the patient then developed fever, cough, and hemoptysis. Chest CT showed bilateral ground‐glass opacities (GGOs) (Fig 3c). Although empirical antibiotics and methylprednisolone (120 mg/day) were administered, the patient did not improve. On admission, PaO_2_ was 58 mmHg. Serum (1‐3)‐ß‐d‐glucan test was 277.7 pg/ml, and serum CMV‐DNA was 31000 copies/mL. BALF NGS was positive for *Pneumocystis jirovecii* and CMV DNA. TMP‐SMX (20 mg/kg/day), intravenous methylprednisolone (40 mg/day), and ganciclovir (5 mg/kg, per 12 hours) were given. Meanwhile, intravenous immunoglobulin (10 g/day for five days) was administered. However, the patient deteriorated progressively. Repeat CT scans indicated the presence of extensive GGOs and multifocal consolidations bilaterally (Fig 3d). Two weeks after admission, he was intubated and died of respiratory failure.

### Case 4

**Figure 4:**
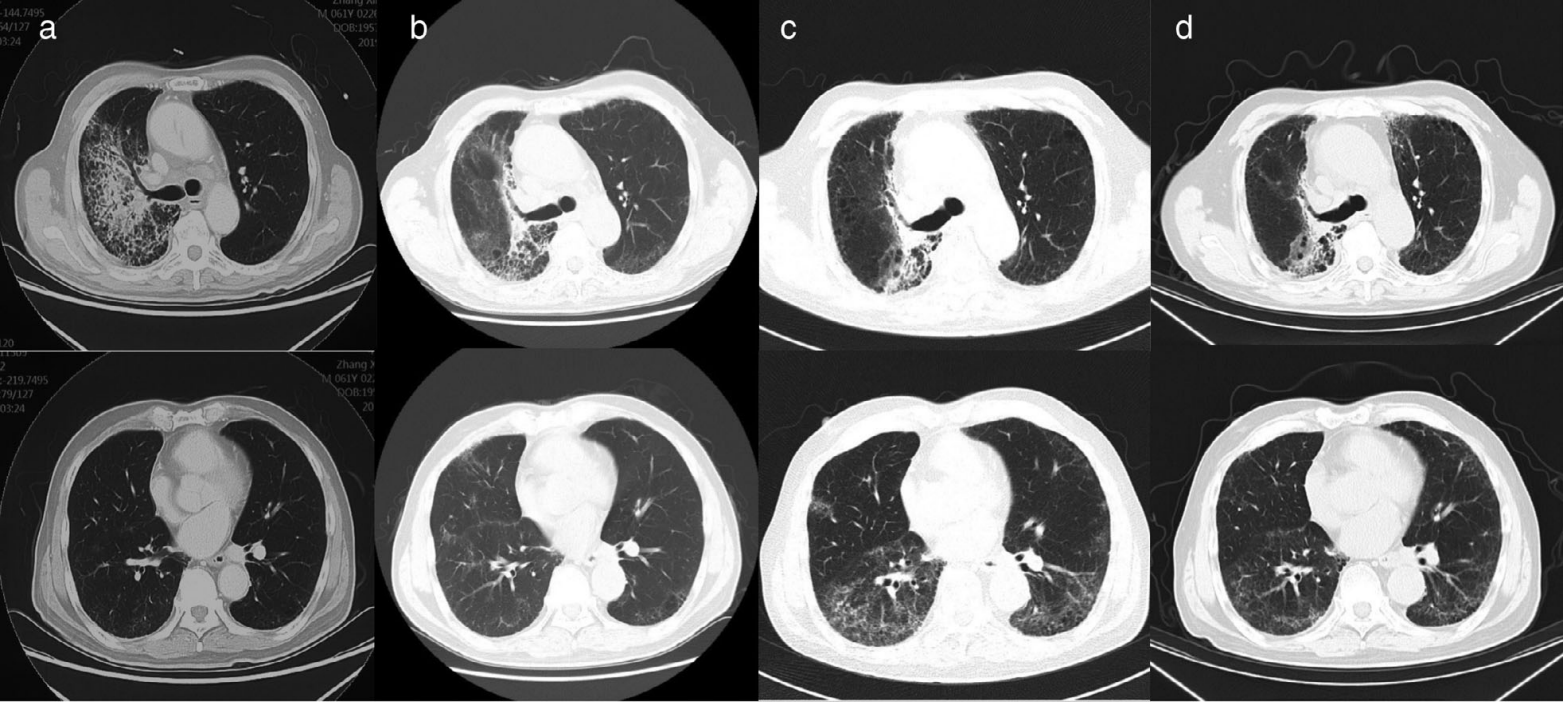
Computed tomography (CT) scan findings in Case 4. (**a**) The patient developed progressive dyspnea and CT scan showed multiple patchy infiltration. (**b**) Dyspnea improved after steroid treatment. (**c**) Scattered ground‐glass opacities (GGOs) with a reticular pattern were evident when the patient developed fever, dyspnea, and productive cough. (**d**) Infiltration resolved after treatment.

A 62‐year‐old male with advanced squamous cell lung carcinoma presented with fever and productive cough for two days. He was treated with two cycles of etoposide and cisplatin, and concurrent radiotherapy was performed. The patient obtained partial remission, and was transitioned to two cycles of toripalimab (PD‐1 inhibitor; 240 mg). He developed progressive dyspnea 10 days after the last dose of toripalimab.CT scans revealed multiple patchy infiltration on the right lung (Fig 4a). An IrAE was considered likely to have occurred, and he received intravenous methylprednisolone (40 mg/day) as well as empirical antibiotics (ceftazidime, 1 g per 12 hours; moxifloxacin, 0.4 g/day). His steroid dose was tapered within six weeks as his symptoms improved (Fig 4b). However, the patient subsequently developed fever, dyspnea, and productive cough after ceasing steroid treatment. CT scan showed scattered GGOs in a pattern of reticular opacities (Fig 4c). BALF lymphocytes percentage were 6%. BALF pathogen culture was negative, while NGS revealed *Pseudomonas aeruginosa*, *Pneumocystis jirovecii*, and *Candida albicans*. Intravenous moxifloxacin (0.4 g/day), fluconazole (200 mg/day), oral SMZ‐TMP (20 mg/kg/day), and intravenous methylprednisolone (40 mg per 12 hours) were administered. The patient's fever subsided, and the dyspnea was gradually relieved. Repeated CT scans showed the infiltration had resolved (Fig 4d).

## Discussion

Opportunistic infections accompanying immunotherapy are severe and can often be fatal. For patients with NSCLC, infections should be carefully distinguished from checkpoint inhibitor pneumonitis (CIP), pseudoprogression, and hyperprogression. Fever is a strong indicator of infection. However, it often requires intensive investigation owing to heterogeneity on clinical presentation.

Two of the above cases did not receive antecedent glucocorticoid therapy, yet developed immune‐related, steroid‐free infection. Similarly, previous reports have described tuberculosis in patients with no previous use of immunosuppressive agents.^4^, ^5^ Although random control trials of CTLA‐4 and PD‐1/PD‐L1 blocking agents did not confirm an independent relationship of ICIs with infection,^6^, ^7^ we do see patients treated with ICIs who suffer from severe infections. Multiple predisposing factors for infection often coexist in patients who receive ICIs, including cytotoxic chemotherapy, radiotherapy, and associated immunosuppressive comorbidities. Noticeably, both of the above two cases had diabetes mellitus. In a previous cohort published by Fujita *et al*.^2^ 32 out of 167 (19.6%) patients with NSCLC who received nivolumab developed infectious diseases, and diabetes was identified as an independent risk factor for infection. We therefore recommend a careful evaluation of patients with diabetes. Further research is needed to better understand the mechanism of increased risk of infection accompanying immunotherapy.

Two of the four patients in our study developed immune‐related, steroid‐induced pneumonia. Current guidelines recommend 1–2 mg/kg/day of prednisone/equivalent glucocorticoids for lower‐grade (ie, grade 2) CIP, and 2–4 mg/kg/day for higher‐grade (ie, grade 3–4) CIP.^8^ In a recent retrospective study involving 740 patients with melanoma treated by ICIs, the rate of serious infection was 13.5% in the subgroup of patients exposed to glucocorticoids or infliximab.^9^ When suspected, opportunistic infections should be thoroughly screened, including *Aspergillus fumigatus* pneumonia, PCP, tuberculosis, and viral infections.

Bronchoscopy and bronchoalveolar lavage (BAL) are essential to confirm an infection. An increased lymphocytic infiltration supported CIP.^10^ In a 2017 case series, 68% (24/35) of CIP cases had T lymphocytic alveolitis.^11^ In our clinical cases, percentages of lymphocytes in BALF were low, suggesting infection rather than irAE. Given the unfavorable effect of steroids on controlling infections, it is important to carry out bronchoscopy BAL in a timely manner in patients with suspected CIP before significant supplemental oxygen requirements are needed.

NGS opens up revolutionary opportunities in diagnostics of infectious diseases. It is especially useful for cases that challenge the limits of traditional laboratory testing.^12^ A 2017 prospective study involving 101 immunocompromised adults showed that NGS had a high negative predictive value, and detected more clinically relevant pathogens than conventional microbiological methods.^13^ In our case reports, patients underwent NGS of BALF and obtained revealing results indicating mixed infection. Pathogens included bacteria (Cases 3 and 4), CMV (Case 1), *Pneumocystis jirovecii* (Case 4), and *aspergillus fumigatus* (Cases 1 and 3). To patients who obtained limited results from traditional microbiological culture, NGS of BALF is beneficial in guiding a precise antimicrobial treatment.

The treatment strategies of opportunistic infections complicating immunotherapy have not as yet been defined. Our clinical cases demonstrated rapid courses, and early diagnosis was pivotal. We also suggest that an antipneumocystis prophylaxis may be considered in selected patients. Prospective trials are required to investigate the optimal procedures and therapeutic management.

## Disclosure

The authors report that there are no conflict of interests.
